# Strategies for Regulating the Color, Texture and Flavor Characteristics of Fermented Meat Products by *Lactiplantibacillus plantarum* and *Saccharomyces cerevisiae*

**DOI:** 10.3390/foods15101750

**Published:** 2026-05-15

**Authors:** Xiang Li, Shouwei Wang, Hao Zou, Shuangli Du, Yingying Li, Jiapeng Li, Mingxuan Liu, Hua Bai, Haitang Wang, Xi Chen

**Affiliations:** 1 China Meat Research Centre, Beijing 100068, China; lx19950606@163.com (X.L.); cmrcwsw@126.com (S.W.); 13718647994@163.com (H.Z.); ying_sky@126.com (Y.L.); ljp7915@126.com (J.L.); 2 Beijing Academy of Food Sciences, Beijing 100068, China; 3 Technology Innovation Center of Animal-Derived Protein Alternatives, State Administration for Market Regulation, Beijing 100073, China; 4 College of Food Science and Engineering, Shaanxi University of Science & Technology, Xi’an 710021, China; slshuang1020@163.com; 5 College of Food and Biological Engineering, Beijing Vocational College of Agriculture, Beijing 100031, China; lmx20050606@163.com (M.L.); 15901556734@163.com (H.B.)

**Keywords:** *Lactiplantibacillus plantarum*, *Saccharomyces cerevisiae*, culture starter, meat products, quality

## Abstract

The effects of direct inoculation of compound culture starter (*Lactiplantibacillus plantarum* and *Saccharomyces cerevisiae*) on the color, texture, and flavor qualities of fermented pork liver, sausage, and jerky were investigated. The results showed that inoculation with *Lactiplantibacillus plantarum* and *Saccharomyces cerevisiae* could significantly increase the lightness value (L*) and chroma value (C*) values of meat products and promote the color development of products. Meanwhile, it can also improve the tenderness of the products. The post-inoculation fermentation process enhanced the diversity and concentration of flavor compounds, improving the overall flavor of the product, and a total of 11 characteristic flavor compounds were identified. Inoculated fermentation reduced the levels of pentanal and methanethiol, which were unpleasant odor components in pork liver. In addition, inoculation fermentation could reduce the concentration of nonanal, which contributes to rancid fat odor in jerky. Inoculated fermentation enriched the flavor profile of meat products through the dual effects of “removing gamey flavor and enhancing aroma”. The results also showed that, after inoculation, the umami taste of meat products was enhanced, and bitter and sour tastes were inhibited. In conclusion, *Lactiplantibacillus plantarum* and *Saccharomyces cerevisiae* could serve as an excellent culture starter for industrial production, providing a feasible path for green and healthy meat processing.

## 1. Introduction

Driven by global population growth, urbanization, industrialization, and rising per capita income, meat consumption has nearly doubled over the past 50 years [1]. The demand for animal-derived products increased from 229 million tons in 2000 to 465 million tons by 2025 [2]. Offal products, minced meat products, and whole-cut products are typical examples that hold significant market share. However, meat products manufactured using traditional processes usually face quality challenges. For instance, offal products are prone to developing volatile compounds that impart metallic or spoiled odors; minced meat products are susceptible to fat oxidation and spoilage bacteria growth during storage; and whole-cut products often become dry, hard, and tough after processing such as drying. These defects severely impact market acceptance and shelf stability [3]. Furthermore, as living standards rise and health awareness increases, consumer food preferences have evolved from basic sustenance to seeking high-quality, convenient, and quick options. Consequently, demand for premium, easy-to-consume, safe, and healthy meat products continues to grow. The traditional additive-based approach to improving meat quality can no longer meet the demands for developing green, safe, and high-quality meat products. In this context, microbial fermentation emerges as an effective solution [4].

Microorganisms, acting as fermentation agents, can regulate product color, texture, flavor, and safety characteristics through their own physiological metabolism [5]. Lactic acid bacteria are commonly used as fermentation agents in meat product production, capable of improving product color, inhibiting the proliferation of pathogenic and spoilage bacteria, and degrading harmful substances commonly found during fermentation to enhance product safety [6]. Yang et al. [4] inoculated fermented beef products with 10^8^ CFU/g *Lactiplantibacillus plantarum* GY1 (isolated from traditional fermented meat from the plateau region). This not only significantly reduced the levels of nitrosamines (NDMA decreased by 62%) and biogenic amines (total histamine and putrescine decreased by 58%) in the product, but also enhanced the redness value (a*) of the product by 43.5% and improved its color uniformity by 24% through promoting the stable formation of nitrosomyoglobin. Furthermore, Liu et al. [7] inoculated fermented mutton sausages with a composite probiotic starter culture, which significantly inhibited the growth of pathogenic bacteria while effectively reducing the accumulation of biogenic amines such as histamine and putrescine in the product. This fermenting agent also promoted the formation of nitrosomyoglobin, increasing the redness value (a*) of the sausage by 18%. By degrading proteins and lipids, it elevated the relative contents of flavor compounds such as aldehydes and esters, resulting in a more pronounced meat aroma and umami flavor. However, the flavor profile generated by *Lactiplantibacillus plantarum* primarily consisted of acidity derived from lactic acid metabolism. The variety and quantity of volatile flavor compounds produced were limited, resulting in a thin flavor layer. Its flavor richness was typically lower than that of products fermented with compound starter cultures or yeast-containing starters. Wu et al. [8] observed that sausages fermented solely with *Lactiplantibacillus plantarum* produced only lactic acid, lacking the alcohols generated by Saccharomyces cerevisiae and esters produced by molds. This resulted in a monotonous flavor profile prone to sour and astringent off-flavors. The blended fermentation system enhanced meatiness and flavor harmony.

Yeast metabolizes carbohydrates to produce ethanol and other byproducts; ethanol undergoes esterification with fatty acids to form esters that impart fruity aromas to the product. Amino acids released from protein breakdown by proteases can undergo Strecker degradation to form flavor precursors such as aldehydes and ketones. These compounds synergize with other microbial metabolites to create rich, distinctive complex flavors. Simultaneously, Saccharomyces cerevisiae reduces off-flavors caused by excessive fat oxidation [9]. Andrade et al. [10] inoculated dry-fermented sausages with *Debaryomyces hansenii*. By degrading carbohydrates and lipids, this strain significantly increased the levels of characteristic flavor compounds such as ethanol and ethyl acetate, enhancing the product’s rich aroma and ester-like flavor, thereby creating a more complex and full-bodied overall flavor profile. Furthermore, Corral et al. [11] compared sausages made from boar meat and sow meat inoculated with yeast. The boar meat sausages exhibited texture and chewiness comparable to those made from sow meat. Simultaneously, yeast degrades skatole in boar meat sausages, significantly improving overall sensory quality. However, Staniszewski et al. [12] found that *Saccharomyces cerevisiae* produces specific biogenic amines during fermentation, which may adversely affect human health upon ingestion. Single-strain fermentation exhibits functional limitations: *Lactiplantibacillus plantarum* possesses strong acid-producing capacity but generates limited flavor profiles, while Saccharomyces cerevisiae contributes rich aromas but has limited antimicrobial effects. This makes it challenging to achieve synergistic optimization of meat product quality across multiple dimensions such as color, texture, and flavor. Therefore, developing proprietary direct-inoculation fermentation agents based on the functional complementarity of different strains, and systematically investigating their application effects and regulatory mechanisms in various processed meat products, has become a key direction for addressing traditional processing challenges [13].

Pork liver, sausage, and jerky were selected as representative examples of offal products, minced meat products, and whole-cut meat products, respectively, to investigate the application of composite starter cultures in different types of meat products. A direct inoculation compound culture starter based on independent selection comprising *Lactiplantibacillus plantarum* and *Saccharomyces cerevisiae* was applied. By establishing fermentation and control groups, and employing multi-dimensional testing methods including textural analysis, intelligent sensory analysis, gas chromatography–ion mobility spectrometry, and sensory evaluation, the differences in color, texture, flavor, and sensory characteristics were analyzed. The aim is to reveal the regulatory mechanisms of inoculated fermentation on the quality of different meat products, clarify its application advantages in “optimizing color, improving texture, and reducing off-flavors while enhancing aroma,” and provide a theoretical basis and practical support for the precise application of direct-inoculation fermentation agents in meat processing. This will promote the industrial upgrading of meat products toward green and healthy processing by reducing reliance on traditional additives [14].

## 2. Materials and Methods

### 2.1. Materials

Pork liver, pork longissimus muscle, pork ham, and pork back fat were all purchased from Beijing Ershang Meat Food Group Co., Ltd. (Beijing, China). Salt, sucrose, glucose, Chinese Baijiu, and various spices were purchased from local supermarkets. Analytical-grade potassium chloride and tartaric acid were purchased from (Sinopharm Chemical Reagent Co., Ltd., Shanghai, China). Reference standards (including 2-methyl-3-heptanone, 2-butanone, 2-pentanone, 2-hexanone, 2-heptanone, 2-octanone, and 2-nonanone) were purchased from Aladdin Biochemical Technology Co., Ltd. (Shanghai, China). YPD medium and MRS medium were purchased from Beijing Luqiao Biotechnology Co., Ltd. (Beijing, China). *Lactobacillus plantarum* CMRC 19L and *Saccharomyces cerevisiae* CMRC Y70 were provided by China Meat Research Center (Beijing, China).

### 2.2. Instruments and Equipment

The EJAD 119 mixer was purchased from Aiko Engineering Co., Ltd. (Nagoya, Japan). The OSCAR20 sausage stuffer was purchased from Heinrich Frey Maschinenbau Co., Ltd. (Baden‑Württemberg, Germany). The meat grinder was purchased from Hobart Deutschland Co., Ltd. (Hamburg, Germany). The BK-56F steam oven was purchased from Bakoln Co., Ltd. (Hamburg, Germany). The KMF 720 constant temperature and humidity chamber was purchased from Binder Co., Ltd. (Hamburg, Germany). The JT-D homogenizer was purchased from Jintian Testing Equipment Research Institute (Luohe, China). The CR-400 colorimeter was purchased from Konica Minolta Investment Co., Ltd. (Tokyo, Japan). The SA-402B flavor analysis system was purchased from Insent Co., Ltd. (Kawasaki, Japan). The TA.XT.plus texture analyzer was purchased from Stable Micro Systems Co., Ltd. (Godalming, UK). The FlavourSpec^®^ gas chromatography–ion mobility spectrometer was purchased from G.A.S. Co., Ltd. (Dortmund, Germany). The CTC-PAL3 static headspace autosampler was purchased from CTC Analytics AG (Zwingen, Switzerland). The MXT-wax capillary column (30 m × 0.53 mm, 1.0 μm) was purchased from Restek Co., Ltd. (Bellefonte, PA, USA).

### 2.3. Method

#### 2.3.1. Preparation of Direct-Inoculation Culture Starter

The direct-inoculation culture starter was prepared according to previous studies with slight modifications [15,16,17]. *Saccharomyces cerevisiae* CMRC Y70 [15] was cultured in YPD medium (28 °C, 24 h), while *Lactiplantibacillus plantarum* CMRC 19L [16] was cultured in MRS medium (30 °C, 24 h). Following expansion, the culture was centrifuged (8000 r/min, 4 °C, 15 min) to harvest the cell pellet. The pellet was washed twice with sterile physiological saline, resuspended in a preservative solution (comprising 10% trehalose, 6% sucrose, and 10% skim milk), lyophilized, and stored at −80 °C. Based on previous studies, a powder concentration of 1.0 × 10^6^ CFU/g [17] yielded optimal addition rates, cost efficiency, and fermentation performance. *Saccharomyces cerevisiae* CMRC Y70 and *Lactiplantibacillus plantarum* CMRC 19L were mixed at a mass ratio of 10:1 (*w*/*w*), activated at 30 ± 2 °C for 30 min, and diluted to 1.0 × 10^6^ CFU/mL. The mixture was then lyophilized and stored at −80 °C until further use. Prior to use, the inoculum was equilibrated at room temperature for 30 min.

#### 2.3.2. Grouping

Six experimental groups were categorized as follows: control pork liver (NG), fermented pork liver (YG), control sausage (NC), fermented sausage (YC), control jerky (NF), and fermented jerky (YF). Fermentation conditions were maintained at 30 °C and 90% relative humidity. The pH of the NC group was adjusted using lactic acid, while all other processing parameters (including temperature, duration, and additive quantities) remained consistent across all groups.

#### 2.3.3. Processing

##### Processing Flow, Operational Procedures, and Formula for Pork Liver

Processing Flow

Raw materials → Trimming → Soaking → Fermentation → Blanching → Preparation of the braising liquid → Braising → Steeping → Drying → Packaging → Pasteurization → Cooling → Final product.

Operational Procedures

a. Raw material: Fresh chilled pork liver was utilized.

b. Trimming: Excess fat and connective tissue were removed.

c. Soaking: The pork liver was soaked under running tap water for 30 min to remove residual blood.

d. Fermentation (omitted for the NG group): The compound culture starter and glucose were dissolved in distilled water. The pork liver was then added, and the container was sealed and placed in a constant temperature and humidity chamber (30 °C, 90% RH). Fermentation was terminated once the pH reached 5.3.

e. Blanching: The pork liver was placed in a pot filled with cold water, which was then heated and maintained at 70–75 °C for 5 min. The pork liver was then removed, rinsed with water at 70–75 °C, and immersed in water at 60 °C for 30 min.

f. Preparation of the braising liquid: The spices were weighed and wrapped in cheesecloth, which was then tightly secured with cotton string. The spice bundle was placed in boiling water and simmered for 30 min to prepare the braising liquid.

g. Braising: Salt, sucrose, Chinese Baijiu, and the blanched pork liver were added to the braising liquid, which was brought to a boil and simmered for 30 min.

h. Steeping: The heat was turned off, and pot was covered. The pork liver was then left to steep in the braising liquid overnight for flavor equilibration.

i. Drying: The pork liver was removed from the braising liquid and placed in a smoker to air-dry at 20 °C with the airflow set to high for 5 min.

j. Packaging: The pork liver was vacuum-packaged in nylon/polypropylene (NY/PP) composite bags.

k. Pasteurization: The packaged pork liver was immersed in water at 80–85 °C for 30 min to conclude the process.

l. Cooling: The packaged pork liver was immediately immersed in ice water to rapidly reduce the temperature.

m. Final product: The packaged pork liver was subsequently stored at 4 °C for further analytical testing and sensory evaluation.

Formulas

Fermentation formula (based on the weight of fresh chilled pork liver): direct-inoculation compound fermentation starter (1%, *w*/*w*), glucose (0.2%, *w*/*w*), and distilled water (100%, *w*/*w*).

Braising formula (based on the weight of blanched pork liver): salt (5%, *w*/*w*), sucrose (3%, *w*/*w*), Chinese Baijiu (1.5%, *w*/*w*), and water (200%, *w*/*w*).

##### Processing Flow, Operational Procedures, and Formula for Sausage

Processing Flow

Raw materials → Trimming → Grinding →Mixing → pH adjustment → Stuffing → Fermentation → Drying → Packaging → Final product.

Operational Procedures

a. Raw materials: Fresh chilled pork ham was utilized.

b. Trimming: Excess fat and connective tissue were removed. Both pork ham and pork back fat were cut into strips for grinding.

c. Grinding: The pork ham and pork back fat was ground separately through an 8 mm die.

d. Mixing: The ingredients were accurately weighed and thoroughly mixed with the ground meat and back fat (excluding the direct-inoculation compound fermentation starter for the NC group).

e. pH adjustment (NC group only): Food-grade lactic acid was added to the mixture to reduce the pH to 5.3.

f. Stuffing: The mixture was stuffed into 32 mm pork casings and linked at 15 cm intervals.

g. Fermentation (YC group only): The sausages were hung in a constant temperature and humidity chamber at 20 °C and 95% RH until the pH dropped to 5.3.

h. Drying: The sausages were dried for 2 months at 12 °C and 85% RH.

i. Packaging: The casings were removed, and the sausages were vacuum-packaged in nylon/polypropylene (NY/PP) composite bags.

j. Final product: The packaged sausages were stored at 4 °C for further analytical testing and sensory evaluation.

Formulas

Sausage formula (based on the combined weight of pork ham and pork back fat at a 8:2 ratio, *w*/*w*): direct-inoculation compound fermentation starter (1%, *w*/*w*), glucose (0.2%, *w*/*w*), salt (3%, *w*/*w*), sucrose (2%, *w*/*w*), curing salt (0.24%, *w*/*w*), and smoked sweet paprika powder (2%, *w*/*w*).

##### Processing Flow, Operational Procedures, and Formula for Jerky

Processing Flow

Raw materials → Thawing → Trimming → Slicing → Mixing → Fermentation → Baking → Cooling → Packaging → Final product.

Operational Procedures

a. Raw materials: Frozen pork loin was utilized.

b. Thawing: The frozen pork loin was thawed slowly at 4 °C until the core temperature reached 0–4 °C.

c. Trimming: Excess fat and connective tissue were removed from the pork loin.

d. Slicing: The pork loin was sliced into 5 mm-thick pieces against the grain.

e. Mixing: All other ingredients were accurately weighed and thoroughly mixed with the pork slices (except for the direct-inoculation compound fermentation starter and glucose, which were omitted for the NF group).

f. Fermentation (omitted for the NF group): The pork slices were placed in a constant temperature and humidity chamber (20 °C, 95% RH) until the pH dropped to 5.3.

g. Baking: The pork slices were spread on drying racks and placed in an oven and baked at 180 °C for 10 min. After being flipped, the slices were baked for an additional 5 min at the same temperature.

h. Cooling: The pork jerky was removed from the oven and cooled to room temperature in a cool, well-ventilated area.

i. Packaging: The pork jerky was cut into 5 cm × 5 cm squares and vacuum-packaged in nylon/polypropylene (NY/PP) composite bags.

j. Final Product: The packaged pork jerky was stored at 4 °C for further analytical testing and sensory evaluation.

Formulas

Jerky formula (based on the weight of pork loin): direct-inoculation compound fermentation starter (1%, *w*/*w*), glucose (0.2%, *w*/*w*), salt (0.7%, *w*/*w*), sucrose (8%, *w*/*w*), oyster sauce (3%, *w*/*w*), light soy sauce (0.6%, *w*/*w*).

### 2.4. Experimental Methods

#### 2.4.1. Color Difference Measurement

Following the method specified in GB/T 7921-2008 Uniform Color Space and Color Difference Formulae [18], the six treatment groups were placed at room temperature (22 ± 1 °C) for 30 min. The colorimeter was calibrated before conducting the measurements along with the color standards. Surface and cross-section color differences were measured for pork liver, cross-section color differences for sausage, and surface color differences for jerky. This yielded values for lightness (L*), redness/greenness (a*), yellowness/blueness (b*), and chroma (C*). C* serves as the key indicator describing color vividness in color difference measurements: higher C* values indicate more vivid colors and higher saturation; smaller values indicate a color closer to gray with lower saturation [19].

The core calculation formula is based on the a* and b* values in the CIE Lab color space. The C* calculation formula is as follows [20]:

$$C^{*}=\sqrt{{\left(a^{*}\right)}^{2}+{\left(b^{*}\right)}^{2}}$$

In the formula, C* represents the chroma value; a* represents the red-green dimension value; b* represents the yellow-blue dimension value.

#### 2.4.2. Texture Testing

##### Shear Force

Following the method of Mattie et al. [21] with modifications, 1 cm surface layers were removed from pork liver and sausage samples. The central portion was selected and trimmed into 1 cm × 1 cm × 2 cm strips for testing. For jerky, a 1 cm wide strip from the middle section was used for testing. The Warner Bratzler blade from the HDP/BS testing accessory was employed. Instrument parameters were set as follows: pre-test and test speed of 1.00 mm/s, post-test speed of 5.00 mm/s.

##### Texture Profile Analysis

Following the method of Herro et al. [22] with modifications, 1 cm surface layers were removed from the periphery of the pork liver and sausage samples. The central portion was selected and trimmed into 1 cm × 1 cm × 1 cm cubes, and subjected to testing. For jerky, a 1 cm × 1 cm sample from the center was tested. A column-type probe P50 was used with the following parameters: pre-test speed 2.00 mm/s, test speed 1.00 mm/s, post-test speed 1.00 mm/s, compression ratio 50%, interval between two compressions 5.00 s, load force 5.00 g.

#### 2.4.3. Sensory Evaluation

Sensory evaluation was conducted for fermented and unfermented pork liver, sausages and jerky. In compliance with the “Ethics Review Measures for Life Sciences and Medical Research In-volving Humans” issued by the National Health Commission of China in 2023 (Document No. 4), specifically Article 32, this study did not collect sensitive personal data or involve commercial interests. All participants were adults, and their data were anonymized to ensure privacy. Participants were fully informed of the study and provided written informed consent prior to participation, with the right to withdraw at any time without penalty. In accordance with the Food Safety Law of the People’s Republic of China, all fermented and unfermented pork liver, sausages and jerky used in the sensory evaluation complied with national food safety standards. It was ensured that no toxic or harmful substances exceeded permissible limits, thereby guaranteeing participant safety at source. In accordance with the ‘Guidelines for Ethical Review of Human Consumption Trials of Health Foods (2023 Edition)’, this study did not constitute a clinical trial of health foods and was exempt from ethical review. The study presented no potential risk to participants. Furthermore, formal ethical approval was not required as the study focused exclusively on the sensory properties of food, a category typically excluded from such mandates.

Adapted from Mrlein et al. [23], all participants voluntarily signed an informed consent form prior to the assessment. Before the evaluation, participants underwent a specialized sensory screening to test their ability to identify basic tastes. The minimum concentrations for the trained panel were adjusted to fall between half the threshold and the threshold itself. In a blind test, participants were provided with 30 mL aqueous solutions of each basic taste (sweet, salty, sour, bitter, and umami) along with a tasteless control (distilled water). Participants were required to identify taste type of each sample or determine whether it was water. The test proceeded in the following order: subthreshold concentration, threshold concentration, and twice the threshold concentration. The screening criterion for panel inclusion was a correct identification rate of at least 70%. Ten experts evaluated five aspects of the pork liver, sausage, and meat jerky: appearance/color, texture, aroma/flavor, mouthfeel, and overall quality. For fermented samples, the aroma/flavor dimension included specific classifications: fermented pork liver (improved fishy/bitter taste), fermented sausage (fermented aroma/flavor harmony), and fermented meat jerky (fermented aroma/flavor harmony). Each aspect was scored out of 10 points, with a maximum total of 60 points. Specific evaluation criteria were as follows: fermented sausage focused on off-odors, and fermented jerky emphasized fermented flavor harmony. Sensory evaluation is necessary and irreplaceable because it can directly determine the acceptability of products. However, it should be noted that the sensory evaluation was conducted with a relatively small group of panelists, which may limit the generalizability of the sensory results. Specific evaluation criteria are as follows Table 1:

**Table 1:** Sensory evaluation score sheet.

Product Categories	Evaluation Dimensions	Specific Evaluation Indicators	Grading Criteria (10-Point Scale)
pork liver	Appearance and Color	Overall color	10 points: Even color with a natural, healthy glow; 8–9 points: Fairly even, slightly dull; 6–7 points: Uneven, darkened; ≤5 points: Dull/burnt.
	Organizational Status	Density and elasticity	10 points: Firm and elastic, springs back instantly when pressed; 8–9 points: Fairly firm with good rebound; 6–7 points: Slightly soft with slow rebound; ≤5 points: Loose and non-rebounding.
	Aroma and Flavor	Improvement in fishy and bitter taste	10 points: No liver-like bitterness or unpleasant taste, only the characteristic aroma of pork liver remains; 8–9 points: Extremely faint bitterness or unpleasant taste; 6–7 points: Noticeable bitterness or unpleasant taste; ≤5 points: Strong bitterness or unpleasant taste.
	Aroma and Flavor	Layers of Fermented Flavor	10 points: Mellow acidity + rich umami, layered complexity; 8–9 points: Fairly balanced, shallow layers; 6–7 points: Overpowering fermentation notes; ≤5 points: Rancid taste.
	Texture and mouthfeel	Smoothness of chewing	10 points: Soft and sticky, easy to chew, does not stick to teeth; 8–9 points: Relatively smooth; 6–7 points: Slightly hard/sticky to teeth; ≤5 points: Too hard to chew/extremely sticky to teeth.
	Comprehensive Advantages	Advantages of Compound Fermentation Agents	10 points: Optimal improvement in flavor, texture, and off-flavor; 8–9 points: Two distinct advantages; 6–7 points: One significant advantage; ≤5 points: No advantages.
Sausage	Appearance and Form	Intestinal fullness	10 points: Plump with no wrinkles, flat cross-section; 8–9 points: Fairly plump, slight wrinkling; 6–7 points: Noticeable wrinkling; ≤5 points: Severe wrinkling/damage.
	Tissue texture	Slice integrity	10 points: Slices are intact with no crumbs or holes; 8–9 points: Relatively intact with minor crumbs; 6–7 points: Numerous crumbs; ≤5 points: Prone to breaking.
	Aroma and Flavor	Fermentation-specific flavor	10 points: Rich meat aroma + subtle fermented acidity, full-bodied and harmonious; 8–9 points: Strong flavor profile; 6–7 points: Mild flavor; ≤5 points: Lacking distinctive flavor characteristics.
	Aroma and Flavor	stench of corruption	10 points: No rancid taste whatsoever; 8–9 points: Very faint off-flavor; 6–7 points: Noticeable off-flavor; ≤5 points: Strong rancid taste.
	Texture and mouthfeel	Juicy	10 points: Juicy and succulent when chewed; 8–9 points: Fairly juicy; 6–7 points: Somewhat dry; ≤5 points: Dry and lacking juice.
	Comprehensive Advantages	Advantages of Compound Fermentation Agents	10 points: Excellent in fullness, flavor, and preservability; 8–9 points: Excellent in two aspects; 6–7 points: Excellent in one aspect; ≤5 points: No advantages.
jerky	Appearance and Color	Surface gloss	10 points: Oily and rosy, with lasting luster; 8–9 points: Fairly bright; 6–7 points: Slightly dull; ≤5 points: Lacking luster/dull and grayish.
	Organizational Structure	Structural compactness	10 points: Dense and void-free, no fragmentation; 8–9 points: Relatively dense, with minor voids; 6–7 points: Numerous voids; ≤5 points: Loose and friable.
	Aroma and Flavor	Richness of Meat Flavor	10 points: Rich meat aroma, no off-flavors; 8–9 points: Fairly rich; 6–7 points: Fairly light; ≤5 points: No meat aroma/off-flavors
	Aroma and Flavor	Harmonization of Fermentation Flavors	10 points: Subtle acidity harmonizes with meat flavor without harshness; 8–9 points: Fairly balanced; 6–7 points: Overpowering acidity; ≤5 points: Rancid taste.
	Texture and mouthfeel	Appropriate firmness	10 points: Soft yet chewy, neither hard nor sticky; 8–9 points: Too tough/too sticky; 6–7 points: Slightly hard/slightly soft; ≤5 points: Too hard/too sticky.
	Comprehensive Advantages	Advantages of Compound Fermentation Agents	10 points: Excellent in fullness, flavor, and preservability; 8–9 points: Excellent in two aspects; 6–7 points: Excellent in one aspect; ≤5 points: No advantages.

#### 2.4.4. Taste Analysis Measurement

The electronic tongue used in this study features probes constructed from various materials, allowing for the analysis of distinct taste profiles. Modified from the method of Shishkanova et al. [24], 10 ± 0.1 g of crushed and homogenized sample was mixed with 100 mL of distilled water and further homogenized until smooth. The mixture was centrifuged at 11,600× *g* for 10 min at 4 °C, then filtered through medium-speed qualitative filter paper to yield at least 60 mL of supernatant. A reference solution containing 30 mM KCl and 0.03 mM tartaric acid was employed as a tasteless control. Sample detection and washing times were set at 120 s and 10 s, respectively. The system was equipped with five sensors: umami, astringency, saltiness, sourness, and bitterness. Based on taste thresholds (e.g., −13 for sourness and −6 for saltiness), data falling below these defined limits were excluded from the analysis [25].

#### 2.4.5. Olfactory Analysis Determination

Following the method of Munekata et al. [26] with modifications, 1.0 ± 0.1 g of the crushed and homogenized sample was accurately weighed and equilibrated at 25 °C for 1 h. The sample was then incubated at 50 °C for 180 s. The electronic nose parameters were set to an injection flow rate of 300 mL/min, a sensor zeroing time of 10 s, and an instrument equilibration time of 180 s. Total signal acquisition lasted 90 s, with sensor readings stabilizing after 60 s. The signal intensity at the 70th second was selected for further data analysis.

#### 2.4.6. Headspace Gas Chromatography-Ion Mobility Spectrometry (HS-GC-IMS)

##### HS-GC-IMS

The GC-IMS (Flavor Spec^®^, G.A.S. Dortmund, Dortmund, Germany) was utilized to determine the volatile flavor headspace compounds in samples. A total of 2.0 g of each sample was weighed into a 20 mL headspace vial [27]. Three parallel determinations were performed for each sample. As an internal standard, 10 μL of 2-methyl-3-heptanone (10 ppm) was added to each vial. The headspace injection conditions were as follows: an incubation temperature of 60 °C for 15 min at a rotation speed of 500 r/min; an injection volume of 200 µL using the splitless mode; and an injection needle temperature maintained at 85 °C.

The GC analysis was performed under the following conditions: the column temperature was maintained at 60 °C with an inlet temperature of 80 °C. High-purity nitrogen (≥99.999%) served as the carrier gas. The programmed flow ramp was initiated at 2.0 mL/min for 2 min, increased linearly to 10.0 mL/min over 8 min, then to 100.0 mL/min over 10 min, and finally reached 150.0 mL/min over the last 10 min. The total chromatographic run time was 30 min.

The IMS conditions were as follows: a tritium (3H) ionization source was used with an ionization tube length of 53 mm. The electric field strength was maintained at 500 V/cm, and the ionization tube temperature was set to 45 °C. High-purity nitrogen (purity ≥ 99.999%) served as the drift gas at a constant flow rate of 75 mL/min. All measurements were conducted in positive ion mode.

##### Data Analysis

Data were analyzed using instrumental analysis software, including Libraries search, Reporter plug-in, and Gallery Plot plug-in, to perform sample analysis from multiple perspectives. The identified compounds were combed for retention index (RI) and drift time (DT) using the G.A.S.’s IMS database search software (G.A.S. version 2.0.0. Dortmund, Germany). The specific steps were as follows: Retention index (RI) comparison—experimental linear RIs were calculated by analyzing n-alkane standard mixtures (2-butanone, 2-pentanone, 2-hexanone, 2-heptanone, 2-octanone and 2-nonanone) under identical chromatographic conditions, and qualitative analysis was accomplished by matching and comparing the acquired data with the NIST 2020 GC retention index library and the built-in IMS migration time database of Vocal software, with deviations within ±20 considered as matches.

The internal standard semiquantitative method was used to calculate the relative concentration of each volatile component according to the compound and internal standard compound peak area ratio. According to the report of Emma et al. [28], the level of confidence for the identified volatile compounds is level 2a (High-confidence level). A variable importance for the projection plots (VIP) was used to evaluate the characteristic flavor of meat products, in which a VIP > 1 indicates the volatile has crucial contributions to the overall flavor, and combined with SIMCA 14.1 was utilized to conduct partial least squares discriminant analysis (PLS-DA) and calculate VIP values.

### 2.5. Statistical Analysis

Three independent batches of samples (replicates) were prepared, and all measurements were conducted in triplicate (triplicate observations) for each batch. Data analysis was performed by using IBM SPSS 25 (IBM SPSS Software, Chicago, IL, USA) (Analytical Software, St Paul, MN, USA) and the results were expressed as the mean values ± standard deviation (SD). Significant differences between the means (*p* < 0.05) were confirmed by a one-way analysis of variance (ANOVA) with Tukey’s multiple comparisons. Origin 2018 (Origin Labs, Hampton, MA, USA) was adopted for graph plotting, while Vocal data processing software (0.4.03, G.A.S. GmbH, Dortmund, Germany) combined with SIMCA 14.1 was utilized to conduct partial least squares discriminant analysis (PLS-DA) and calculate variable importance in projection (VIP) values.

## 3. Results and Analysis

### 3.1. Color Difference Analysis

**Figure 1:**
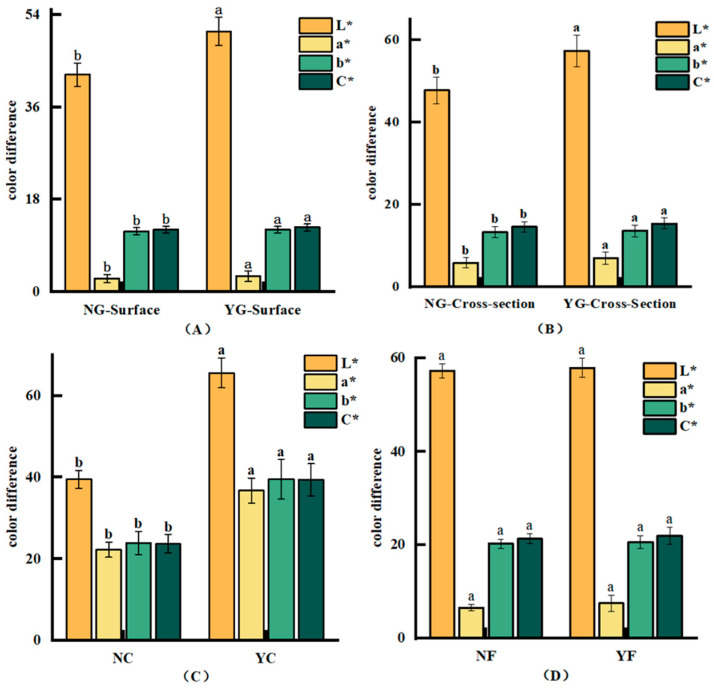
Color difference analysis. (**A**): Color variation on the cross-section of pork liver; (**B**): Color variation on the surface of pork liver; (**C**): Color variation of sausage; (**D**): Color variation of jerky. The significant differences among different treatments are indicated by different lowercase letters (a, b) (*p* < 0.05).

First, it should be noted that the results of this study were obtained using a control group model involving the use of lactic acid in fermented sausages, and therefore had certain limitations. Color serves as a crucial indicator reflecting the edible quality of meat products, representing the most intuitive criterion for consumers to assess their quality [29]. As shown in Figure 1, inoculation with fermentation significantly influenced the L*, a*, b*, and C* values of pork liver and sausage (*p* < 0.05). The L* values of the YG and YC groups were significantly higher than those of the NG and NC groups (*p* < 0.05). Notably, the L* values on the surface and cross-section of fermented pork liver were significantly higher than those of the non-inoculated group. This indicated that inoculation fermentation enhanced brightness, resulting in a brighter product appearance. Simultaneously, enhanced redness values impart a more appealing color to the product, aligning with consumer expectations for high-quality product appearance [30]. The a* values of the YG and YC groups were significantly higher than those of the NG and NC groups (*p* < 0.05). Simultaneously, these groups exhibited higher brightness and saturation, resulting in a more appealing bright red color. Studies have reported that the microorganisms in the inoculated fermentation groups secrete nitrate reductase, promoting nitrosomyoglobin formation and enhancing product redness values [31]. Furthermore, the C* values of the YG and YC groups were significantly higher than those of the NG and NC groups (*p* < 0.05). Consumers typically associate a product’s redness and vividness with its quality and freshness. Based on this, meat products from inoculated fermentation groups were more favored by consumers, indicating that the compound culture starter of *Lactiplantibacillus plantarum* and *Saccharomyces cerevisiae* can enhance product color development.

### 3.2. Texture Analysis

Tenderness is a core indicator for evaluating the edible quality of meat products. It refers to the softness, juiciness, and chewability experienced when consuming meat. Tenderness is typically assessed through three dimensions—ease of biting, degree of chewing, and residual fiber content—and it inversely correlates with meat toughness [32]. Shear force serves as a direct indicator of meat product tenderness, with lower values indicating higher tenderness. Table 2 indicated that inoculation fermentation significantly affected shear force values in fermented pork liver, sausage, and jerky (*p* < 0.05). Shear force values in the NG, NC, and NF groups were significantly higher than those in the YG, YC, and YF groups (*p* < 0.05), demonstrating that inoculation fermentation improves meat product tenderness. This may be due to the synergistic action of enzymes secreted by *Saccharomyces cerevisiae* and *Lactiplantibacillus plantarum* that degrade collagen and actin, reducing the mechanical strength of muscle tissue [33]. This change facilitates the breakage of muscle fibers during chewing, which improves the tenderness of meat products.

As shown in Table 2, the hardness values of the NG, NC, and NF groups were significantly higher than those of the YG, YC, and YF groups (*p* < 0.05). Pork liver often exhibited high hardness and poor elasticity after steaming. In the TPA measurements, the YG group exhibited reduced hardness and chewiness values, while elasticity and cohesiveness values increased (*p* < 0.05). This indicated fermentation effectively mitigates the dry and tough texture defect in cooked pork liver products. These findings were similar to the results of Coelho et al. [34], where fermented meat products inoculated with *Lactobacillus paracasei* and *Saccharomyces cerevisiae* exhibited approximately 40% increased elasticity. Indicators such as hardness and chewiness correlate with muscle tissue density. Jerky exhibits high hardness and poor chewability due to tight protein cross-linking. The reduction in meat firmness may be due to proteolytic degradation during the fermentation process, and the small amount of carbon dioxide produced by yeast metabolism may form microporosity in the meat tissue, thereby reducing the formation of dense protein cross-links [32]. It may also be attributed to *Lactiplantibacillus plantarum*, which lowers water activity at a relatively gentle rate, preventing excessive dehydration that leads to dry, tough meat, and thereby improving the quality of the jerky [4].

### 3.3. GC-IMS Analysis

**Figure 2:**
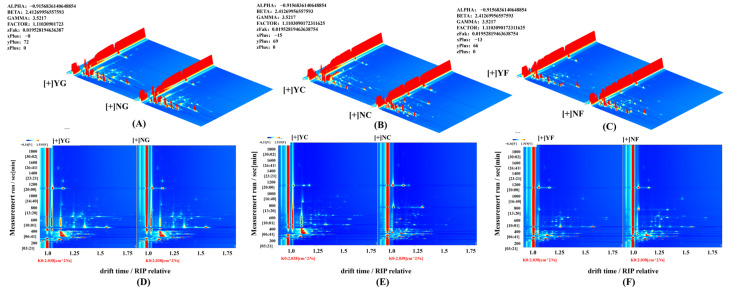
GC-IMS three-dimensional map and difference spectrum. (**A**): Three-dimensional GC-IMS spectrum of volatile components in pork liver; (**B**): Three-dimensional GC-IMS spectrum of volatile components in sausage; (**C**): Three-dimensional GC-IMS spectrum of volatile components in jerky; (**D**): Differential GC-IMS spectrum of volatile components in pork liver; (**E**): Differential GC-IMS spectrum of sausage volatile components; (**F**): Differential GC-IMS spectrum of jerky volatile components.

Gas chromatography coupled with ion mobility spectrometry (GC-IMS) was employed to analyze the differences and changes in volatile components of fermented meat products. The GC-IMS three-dimensional spectra of volatile components in fermented meat products are shown in Figure 2A–C. These maps visually illustrate the differences in volatile organic compounds between inoculated and non-inoculated fermented samples. To further highlight the variations in volatile components, the spectrum of the control sample was selected as a reference. The spectra of other samples were subtracted from this reference, yielding differential comparison maps for different samples, as shown in Figure 2D–F. If the volatile organic compound content in the target sample and the reference is identical, the background after subtraction appears white. Red indicates that the concentration of that substance is higher in the target sample than in the control group, while blue indicates that the concentration is lower in the target sample than in the control group [35]. The figures reveal that the fermented sausage and jerky showed significantly increased flavor compound levels, likely due to microbial metabolic decomposition. This aligns with findings by Kang et al. [6] and Yu et al. [36], where glycolysis and decarboxylation pathways enhanced the flavor compound contents in fermented samples. Increased flavor compound diversity and concentrations contribute to the improved overall flavor in fermented products.

**Figure 3:**
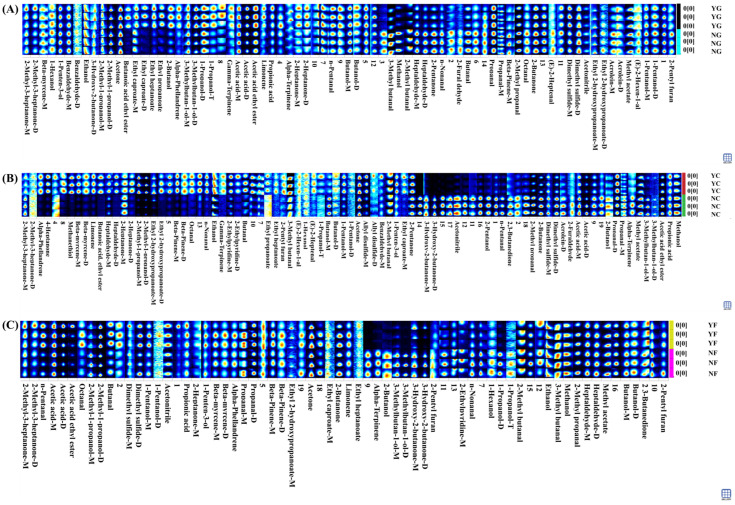
Fingerprints of volatile components. (**A**): Fingerprint diagram of pork liver; (**B**): Fingerprint diagram of sausage; (**C**): Fingerprint diagram of jerky.

To further compare volatile compounds in inoculated and non-inoculated fermented samples, GC-IMS technology effectively separates characteristic volatile components across different samples. A total of 77 flavor compounds were identified in fermented pork liver; 83 flavor compounds in fermented sausage; and 68 flavor compounds in fermented jerky. Fingerprint analysis of all volatile compounds is shown in Figure 3A–C. A total of 48 major flavor compounds were identified in the fingerprint profiles of the NG and YG groups, including β-linalool, n-hexanol, 1-penten-3-ol, benzaldehyde, ethanol, 2-methylpropanol, ethyl hexanoate, ethyl heptanoate, heptanal, 2-pentanone, nonanal, furfural, n-butyraldehyde, propionaldehyde, and 2-pentylfuran. The NC and YC groups exhibited 48 major flavor compounds in their fingerprint spectra, including α-pinene, 4-heptanone, methanethiol, β-linalool, limonene, heptanal, 2-heptanone, 2-methylpropanol, octanal, nonanal, ethanol, 2-pentylfuran, 3-methylbutanal, n-butyraldehyde, and ethyl heptanoate. The NF and YF groups exhibited 42 major flavor compounds in their fingerprint spectra, including pentanal, acetic acid, ethyl acetate, octanal, 2-methylpropanol, n-butyraldehyde, methanethiol, dimethyl sulfide, pentanol, propionic acid, 2-heptanone, 1-penten-3-ol, isobutyraldehyde, heptanal, and methyl acetate.

Flavor compounds identified in fermented meat products include aldehydes, alcohols, esters, ketones, and furans. Aldehydes and alcohols are more abundant in the samples. The primary pathway for aldehyde formation is the oxidation of unsaturated fatty acids, with nonanal contributing a citrus peel aroma. 3-Methylbutanal imparts a malty aroma; methylbutanal contributes both malty and nutty notes, forming a core aroma in roasted meats. However, under microbial action, aldehydes are unstable compounds prone to reduction to alcohols or oxidation to acids [37]. Ethanol contributes to fruit and wine aromas; 3-methylbutanol imparts fruity and malty notes while inhibiting unwanted microbial growth; 1-hexanol imparts a grassy aroma. Dehydrogenation, aldehyde reduction, and hydrolysis of linoleic or linolenic acids during *Lactiplantibacillus plantarum* fermentation are primary drivers of increased alcohol content [16]. Pentalone and 2-heptanone impart fruity and buttery flavors, enhancing the pleasantness of fermented samples. Their increased content stems from enzymatic decarboxylation of carboxylic acids and microbial β-oxidation of lipids such as oils or fats [38]. Acetic acid is the predominant volatile acid in *Lactiplantibacillus plantarum* fermentation broths, generated via the pyruvate–formate cleavage pathway [39]. Acidic compounds influence flavor characteristics by modulating pH and can react with alcohols through esterification to form aromatic esters.

**Figure 4:**
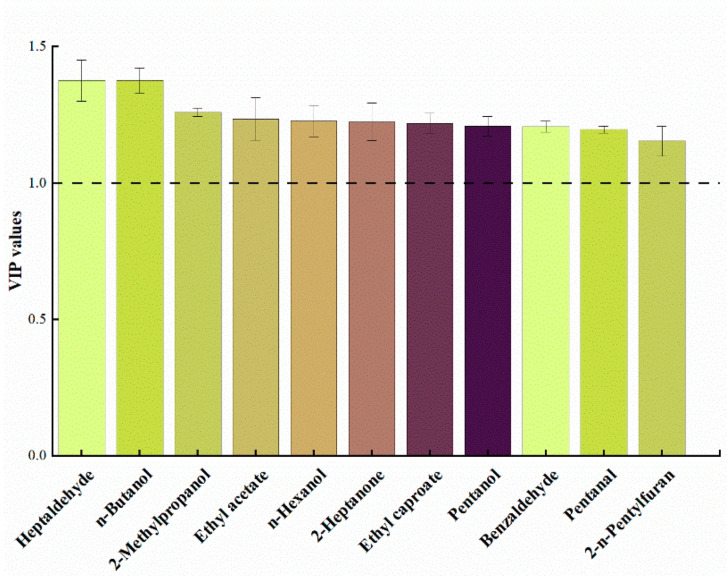
Variable importance projection (VIP) scores.

The VIP value can be used to quantify the contribution of each volatile compound to intergroup differences. A common screening criterion is that when VIP > 1, the volatile compound significantly contributes to intergroup differences and is considered a characteristic compound [40,41,42]. As shown in Figure 4, a total of 11 characteristic flavor compounds were screened, including 4 alcohols, 3 aldehydes, 2 esters, 1 ketone, and 1 furan. These were n-butanol, heptanal, 2-methylpropanol, ethyl acetate, n-hexanol, 2-heptanone, ethyl hexanoate, benzaldehyde, pentanal, pentanol, and 2-pentylfuran. In the YG group, the levels of heptanal (fruity and wine-like aroma) and n-hexanol (sweet aroma) increased by 42.3% and 38.7%, respectively, compared to the NG group. Conversely, the levels of pentanal (grass-like pungent taste) and methanethiol (sulfur odor) decreased by 51.2% and 46.8%, respectively, compared to the NG group. This indicated that inoculated fermentation removed off-flavors from pork liver while increasing aromatic compound contents. *Saccharomyces cerevisiae* reduced α-helix content in pork liver protein, exposing hydrophobic groups that bind to pentanal via hydrophobic interactions, thereby decreasing its headspace concentration [43]. This aligns with the findings of Xue et al. [13] that Saccharomyces cerevisiae fermentation reduced the off-flavor concentrations via protein hydrophobic interactions. In the YC group, ethyl hexanoate (pineapple fruit aroma) and 3-methylbutanol (whiskey aroma) levels increased by 58.5% and 49.1% compared to the NC group, respectively. Lactic acid produced by *Lactiplantibacillus plantarum* served as a carbon source for *Saccharomyces cerevisiae*, promoting ester synthesis, while *Saccharomyces cerevisiae* decarboxylases regulate ketone formation [44]. In the YF group, ethyl acetate (fresh fruit flavor) and heptanoic acid ethyl ester (pineapple aroma) levels increased by 62.4% and 57.8% compared to the NF group, while nonanal (oily flavor) decreased by 48.3%.

### 3.4. Sensory Evaluation Analysis

Following inoculation and fermentation, the starter culture modulates the physicochemical properties of meat products, enhancing color uniformity and surface gloss without compromising product integrity. As shown in Table 3, the overall color scores for the YG, YC, and YF groups were significantly higher than those for the NG, NC, and NF groups (*p* < 0.05). The YG group resolved the dull, dark appearance typical of pork liver; the YC group exhibited an appealing bright red hue; while the YF group maintained a rosy luster. The surface gloss of the YF group was significantly higher than that of the NF group (*p* < 0.05). This was consistent with the color results. The YG group exhibited significantly superior firmness and elasticity compared to the NG group (*p* < 0.05). The YG group overcame the dense and dry texture characteristic of unfermented pork liver. Meanwhile, the YG group maintained its complete shape after cooking, resolving the crumbly crumbling issue encountered during slicing of unfermented products. The masticatory properties of the YG group were significantly better than those of the NG group (*p* < 0.05).The YC group showed no significant difference in sausage plumpness compared to the NC group (*p* > 0.05), indicating that inoculation fermentation does not disrupt meat tissue morphology [45,46], and the juiciness of the YC group was significantly higher than that of the NC group (*p* < 0.05). Additionally, the YF group’s structural density was significantly superior to the NF group (*p* < 0.05).

Post-inoculation fermentation enhances meat product flavor complexity by “reducing off-flavors and enhancing aroma.” The YG group scored significantly higher than the NG group in off-flavor improvement (*p* < 0.05). The inherent off-flavor in pork liver stems from low-molecular-weight fatty aldehydes and similar compounds, which *Lactiplantibacillus plantarum* can degrade, which was consistent with GC-IMS results [47]. The YC group suppressed rancid flavors from fat oxidation through fermentation, consistent with electronic nose detection. The meat aroma in the YC group was significantly superior to that in the NC group (*p* < 0.05). This resulted from *Lactiplantibacillus plantarum* breaking down proteins to produce flavor precursors such as amino acids, while Saccharomyces cerevisiae exhibited strong ester-producing capabilities [48]. No significant difference in meat aroma intensity was observed between the YF and NF groups (*p* > 0.05), indicating that inoculation fermentation enhanced aroma without masking the meat’s inherent flavor profile. In summary, the inoculated and fermented product achieves a “smooth, juicy, and palatable” texture.

### 3.5. Intelligent Sensory Evaluation Analysis

**Figure 5:**
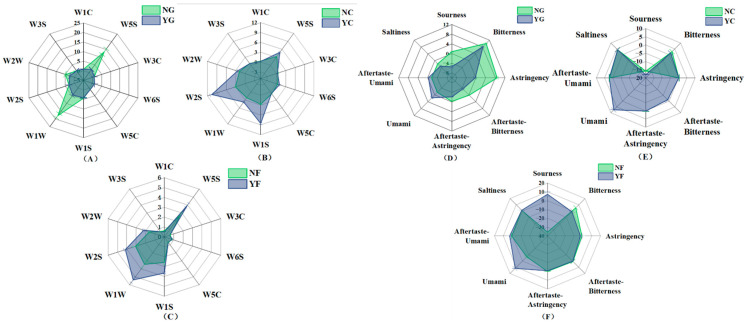
Intelligent sensory analysis. (**A**): Pork Liver Electronic Nose; (**B**): Sausage Electronic Nose; (**C**): Jerky Electronic Nose; (**D**): Pork Liver Electronic Tongue; (**E**): Sausage Electronic Tongue; (**F**): Jerky Electronic Tongue.

The radar chart shown in Figure 5A–C displays the response values of the electronic nose’s 10 odor sensors, representing the concentration of corresponding odorants. The high response value of the W1W sensor in the NG group indicated that sulfur-containing functional groups (organic sulfides) such as sulfhydryl and sulfide bonds in the fishy odor of pork liver were sensitive to this sensor [49]. The W1W sensor response in the YG group was significantly reduced, indicating that inoculation fermentation decomposed the amines and sulfides responsible for the fishy odor in pork liver through microbial metabolism, resulting in a milder sensory profile. Additionally, the low W5S sensor response in the YG group reflected weaker fat oxidation. The elevated responses of W2S and W1S sensors in the YC and YF groups indicated higher levels of aldehydes, ketones, alcohols, and alkanes in the sausages post-inoculation fermentation. Furthermore, the larger olfactory profile area in the YC group suggested that fermentation intensified the meat aroma and introduced additional fermentative notes, enhancing the complexity of the scent profile.

As shown in Figure 5D–F and Table 3, the sensor responses for sourness, bitterness, astringency, bitter aftertaste, and astringent aftertaste in the YG group were all lower than those in the NG group, with particularly pronounced reductions in the astringency and sourness profiles. The sensor responses for sourness and bitter aftertaste in the YC group were both lower than those in the NC group, especially the YC group’s lack of sourness perception being particularly prominent. The umami sensor response values in the YF group were higher than those in the NF group, while the taste sensor response values were lower than those in the NF group. The fermented meat jerky exhibited a uniform flavor profile. This indicated that inoculation fermentation enhanced the umami dimension of the sample, reduced its sourness perception, suppressed bitterness formation, and lowered salt content, bringing the sample’s salt content closer to the palatability range. This result was consistent with the GC-IMS conclusions. Inoculation fermentation achieved “effective enhancement (umami) + neutralization (sour/astringent flavors) + palatability improvement (saltiness/bitterness)” in meat products by regulating taste substance metabolism and selectively degrading undesirable taste components [50], thereby elevating the overall taste experience.

## 4. Conclusions

The results indicated that inoculation with *Lactiplantibacillus plantarum* and *Saccharomyces cerevisiae* significantly enhanced the L* and C* values in meat products, promoting color development. Inoculation fermentation enhanced the tenderness and elasticity while reducing hardness. Post-inoculation fermentation enhanced the diversity and concentration of flavor compounds, improving overall product flavor. Aldehydes and alcohols exhibit increased diversity and abundance, with 11 characteristic flavor compounds identified (VIP > 1), including n-butanol, heptanal, 2-methylpropanol, ethyl acetate, n-hexanol, heptanone, ethyl hexanoate, benzaldehyde, pentanal, pentanol, and 2-pentylfuran. Inoculated fermentation reduced the levels of pentanal and methanethiol—unpleasant odor components in pork liver—and components such as nonanal, which contributes to rancid fat odor in jerky. After inoculation fermentation, electronic nose sensors showed significantly reduced responses to sulfides and amines associated with fishy odors, while responses to aldehydes, ketones, and alcohols linked to meatiness and fermentation aromas increased, resulting in richer flavor complexity. Inoculation fermentation enhanced umami while suppressing bitterness and sourness, bringing saltiness and astringency closer to the palatability range. Sensory evaluation results aligned with color, tenderness, TPA, GC-IMS, electronic nose, and electronic tongue findings, confirming that inoculation fermentation enriched meat product flavor complexity through dual effects of “reducing fishiness and enhancing aroma.” However, this study also had certain limitations, including pH adjustment in the sausage control group using lactic acid and a relatively small group of panelists in the sensory evaluation. This study still indicated that the addition of *Lactiplantibacillus plantarum* and *Saccharomyces cerevisiae* effectively regulated the color, texture, and flavor of fermented meat products, thereby improving their overall edible characteristics. These microorganisms serve as excellent fermentation agents for industrial production.

## Figures and Tables

**Table 2:** Shear force and TPA analysis of pork liver, sausage and jerky.

Sample	Shear Force (g)	Hardness (g)	Adhesiveness (g·s)	Springiness	Cohesiveness	Gumminess	Chewiness	Resilience
NG	2513.67 ± 461.57 ^a^	1746.22 ± 86.81 ^a^	−7.78 ± 0.08 ^a^	0.78 ± 0.01 ^a^	0.58 ± 0.03 ^b^	1384.61 ± 42.31 ^a^	1286.16 ± 56.45 ^a^	0.20 ± 0.00 ^b^
YG	1419.88 ± 69.77 ^b^	1142.88 ± 72.10 ^b^	−1.65 ± 1.12 ^b^	0.82 ± 0.02 ^b^	0.72 ± 0.01 ^a^	821.98 ± 141.68 ^b^	666.62 ± 14.21 ^b^	0.31 ± 0.01 ^a^
NC	2061.34 ± 80.79 ^a^	4358.73 ± 90.71 ^a^	−10.45 ± 6.98 ^a^	0.49 ± 0.02 ^a^	0.32 ± 0.03 ^a^	1417.40 ± 370.76 ^a^	693.48 ± 26.54 ^b^	0.08 ± 0.01 ^a^
YC	1805.74 ± 58.37 ^b^	3851.21 ± 89.17 ^b^	−10.10 ± 6.34 ^a^	0.54 ± 0.02 ^b^	0.34 ± 0.02 ^a^	1488.36 ± 416.94 ^a^	746.39 ± 12.47 ^a^	0.08 ± 0.01 ^a^
NF	684.09 ± 73.02 ^a^	6965.12 ± 180.31 ^a^	−0.30 ± 0.31 ^a^	0.93 ± 0.02 ^a^	0.83 ± 0.02 ^a^	5646.83 ± 702.72 ^a^	5583.30 ± 81.92 ^a^	0.40 ± 0.06 ^a^
YF	525.84 ± 65.99 ^b^	6409.60 ± 165.73 ^b^	−0.92 ± 0.73 ^a^	0.99 ± 0.05 ^a^	0.82 ± 0.03 ^a^	6058.67 ± 1721.42 ^a^	5641.15 ± 169.83 ^a^	0.39 ± 0.07 ^a^

Different letters indicate that significant differences exist within the type of product (*p* < 0.05).

**Table 3:** Sensory scores of meat production.

Evaluation Indicators	NG	YG	NC	YC	NF	YF
Overall color	7.80 ± 0.40 ^b^	9.20 ± 0.80 ^a^	n.d.	n.d.	n.d.	n.d.
Firmness and elasticity	7.60 ± 0.50 ^b^	9.10 ± 0.50 ^a^	n.d.	n.d.	n.d.	n.d.
Improvement in fishy odor	5.80 ± 0.40 ^b^	8.90 ± 0.20 ^a^	n.d.	n.d.	n.d.	n.d.
Layers of Fermented Flavor	n.d.	8.60 ± 0.50	n.d.	n.d.	n.d.	n.d.
Smoothness of chewing	6.60 ± 0.50 ^b^	8.60 ± 0.50 ^a^	n.d.	n.d.	n.d.	n.d.
Intestinal fullness	n.d.	n.d.	8.00 ± 0.70 ^a^	8.80 ± 0.40 ^a^	n.d.	n.d.
Slice integrity	n.d.	n.d.	7.60 ± 0.50 ^a^	8.40 ± 0.50 ^a^	n.d.	n.d.
The distinctive flavor of sausage	n.d.	n.d.	6.80 ± 0.40 ^b^	8.20 ± 0.40 ^a^	n.d.	n.d.
Risk of spoilage (flavor level)	n.d.	n.d.	6.20 ± 0.40 ^b^	8.20 ± 0.40 ^a^	n.d.	n.d.
Juiciness	n.d.	n.d.	5.20 ± 0.40 ^b^	8.60 ± 0.50 ^a^	n.d.	n.d.
Surface gloss	n.d.	n.d.	n.d.	n.d.	6.80 ± 0.80 ^b^	8.80 ± 0.40 ^a^
Structural compactness	n.d.	n.d.	n.d.	n.d.	6.20 ± 0.40 ^b^	8.40 ± 0.50 ^a^
Richness of Meat Flavor	n.d.	n.d.	n.d.	n.d.	7.40 ± 0.50 ^a^	8.00 ± 0.70 ^a^
Harmonization of Fermentation Flavors	n.d.	n.d.	n.d.	n.d.	n.d.	8.20 ± 0.80
Moderate firmness	n.d.	n.d.	n.d.	n.d.	5.40 ± 0.50 ^b^	7.80 ± 0.40 ^a^
Exclusive Advantages of the Multi-Strain Bacterial Agent	n.d.	8.70 ± 0.40	n.d.	8.90 ± 0.50	n.d.	8.80 ± 0.40

n.d.: volatile compounds not detected. Different lowercase letters (a, b) in the same row indicate significant differences among different samples (*p* < 0.05).

## Data Availability

The original contributions presented in the study are included in the article, further inquiries can be directed to the corresponding authors.
